# Trends of Pediatric Anterior Cruciate Ligament Reconstruction Surgery in Korea: Nationwide Population-Based Study

**DOI:** 10.3390/jcm14041389

**Published:** 2025-02-19

**Authors:** Jihyun Hwang, Young-Min Kwon, Collin Lee, Sung Jae Kim, Young-Jin Seo

**Affiliations:** 1 Department of Biomedical Engineering, Johns Hopkins University School of Medicine, Baltimore, MD 21205, USA; jhwang50@jh.edu; 2 Department of Orthopedic Surgery, Hallym University Dongtan Sacred Heart Hospital, Hwaseong 18450, Republic of Korea; kwonym0814@gmail.com; 3 Department of Biology, University of Maryland, College Park, MD 20742, USA; clee1241@terpmail.umd.edu

**Keywords:** ACL injuries, pediatric orthopedics, ACL reconstruction, growth plate, epidemiology, skeletally immature patients, sports injuries

## Abstract

**Background**/**Objective**: The rise in sports participation among children and adolescents has led to an increase in ACL injuries in skeletally immature individuals. This study analyzed nationwide trends in the anterior cruciate ligament (ACL) reconstruction surgeries in pediatric and adolescent populations, addressing concerns about growth plate disturbances with surgery and risks of secondary injuries with nonoperative treatment. **Methods**: We conducted a retrospective population-based cohort study using the National Health Insurance Corporation database in South Korea, analyzing ACL reconstruction trends from 2011 to 2018. Patients were categorized into four age groups (≤12 years, 13–15 years, 16–17 years, and ≥18 years). The Chi-square linear-by-linear association test was used to analyze trends in procedural volumes, age groups, and regions. Poisson regression was used to examine whether differences over time were statistically significant by modeling count data and estimating incidence rate ratios (IRRs) with 95% confidence intervals (CIs). It also evaluates the statistical differences by age, gender, and hospital location. **Results**: A total of 83,132 patients underwent ACL reconstruction during the study period. The ≤12-year-old group accounted for a stable, low percentage of surgeries (16.6% in 2011 to 23.7% in 2018) with no significant trends observed (IRR = 0.99, *p* = 0.683). Conversely, significant increases were noted in patients aged 13–15 years (IRR = 1.04, *p <* 0.001), 16–17 years (IRR = 1.03, *p <* 0.001), and aged ≥18 years (IRR = 1.03, *p <* 0.001). Male patients and urban hospital locations were associated with higher surgical rates. **Conclusions**: During the study period, ACL reconstruction is rare in children ≤12 years old due to concerns about growth disturbances, while adolescents 13–17 years old show increased surgical rates due to evolving practices supporting early intervention. These findings emphasize the need for individualized treatment balancing early benefits and growth plate risks.

## 1. Introduction

Recently, as more pediatrics and adolescents have been participating in youth sports, especially competitive sports, youth sports-related ACL injuries have increased [1]. Several studies reported that ACL injuries in skeletally immature individuals account for a considerably high percentage of contact sports precipitant with knee injuries and adolescent patients with acute knee hemarthrosis [2]. In comparison to the management of a tibial eminence avulsion, it has been a dilemma for orthopedic surgeons to make an optimal treatment decision for skeletally immature patients with ACL ruptures [3].

There have been debates regarding issues between two treatment pathways: secondary chondral and meniscal injuries following nonoperative treatment versus the risk of physeal injuries associated with tunneling across the physis during ACL reconstruction [3]. Patients with a complete or high-grade partial tear of the ACL, who underwent conservative treatment, have a potential possibility of subsequent chondral and meniscal injuries due to persistent instability [4]. In this regard, early ACL reconstruction in skeletally immature patients is rational for restoring ACL function [5,6]. However, conventional ACL reconstruction techniques have an innate problem associated with violating open epiphyseal growth plates [7]. This concern has led to delayed ACL reconstruction protocols until skeletal maturity in some centers [8].

Several reports demonstrated that nonoperative treatment for skeletally immature patients may be a reasonable treatment option for those who can comply with the physical activity restrictions [9]. The above-mentioned conservative treatment of ACL injury may be appropriate in low-level activity patients. However, even with appropriate conservative treatment programs, it has been reported that young active adolescents who have undergone nonoperative treatment often have a concerning rate of subsequent meniscal and chondral injuries, which may potentially result in secondary arthrosis [10]. In this regard, the current trend has led to increased surgical treatment for active pediatric patients with considerable instabilities due to ACL injuries [11]. Beck et al. recently reported an increase in pediatric ACL reconstruction rate over a 20-year period (1994–2013) using data from insurance-serviced individuals [12].

Given these complexities, understanding how the management of ACL injuries in younger patients has evolved over time is essential. Analyzing long-term trends in ACL reconstruction across different age groups can provide valuable insights into evolving treatment strategies, surgical indications, and potential disparities in access to care.

The true trend of ACL reconstruction in the pediatric population remains unknown. Studies evaluating reconstruction incidence trends in children and adolescents on a population basis have been limited in the United States because of a large variety of patient registration systems [13]. Meanwhile, in South Korea, the entire population residing within the territory of Korea is covered by the National Health Insurance (NHI) of Korea as a social insurance benefit system. Hence, using the National Health Insurance Corporation (NHIC) database in South Korea, researchers can analyze all nationwide claims data, including the International Classification of Disease (ICD) codes and information on treatment prescriptions after an official review committee approves [14].

The present study aims to analyze the recent epidemiologic trends of pediatric ACL reconstruction in the pediatric and adolescent populations using the NHIC database in Korea. Although previous studies have examined ACL reconstruction trends in specific age groups, there is limited research analyzing longitudinal changes across all age groups, particularly in pediatric patients. This study aims to fill this gap by providing a comprehensive analysis of ACL reconstruction trends over time. It was hypothesized that the trend in pediatric ACL reconstructions would increase, irrespective of sex and age.

## 2. Materials and Methods

### 2.1. Data Source

In this study, all data were provided by the NHIC database in Korea in January 2020. The information was obtained upon receipt of payment for the data. The NHIC database stores data comprising approximately 97% of the enrolled Korean population who receive a semi-compulsory medical examination biennially.

Basic demographic characteristics, including sex, age, height, and weight, were collected from the national insurance database. International Classification of Diseases, 9th Revision Clinical Modification (ICD-9-CM) diagnostic codes, and details of the treatment prescriptions were also available. The database was queried for a diagnosis of ACL tear (ICD-9-CM code 717.83 or 844.2) from 2009 to 2019. A total of 2,333,478 patients under the age of 45 with diagnostic codes for ACL injuries in their medical records were ultimately included in the current study. Only the first date was included if the patient had more than 1 claim for an ACL reconstruction during the evaluation period.

Personal data provided by the NHIC was de-identified because the whole database was secured by altering the identifiable personal information. To ensure patient confidentiality, direct identifiers such as name, resident registration number, and exact address were removed, while indirect identifiers, including birth year and administrative division codes, were provided in an aggregated form to prevent re-identification. The Institutional Review Board (IRB) of Hallym University Dongtan Sacred Heart Hospital approved the study protocol. Due to the nature of public data from NHIC, no informed consent was required from patients.

### 2.2. Study Design

The present study evaluated a retrospective population-based cohort. The purpose of the current study is to analyze the 10-year surgical trends of ACL reconstruction surgery in age groups with immature growth plates. To begin, the study population was grouped by age: those aged 12 years old and under, representing entirely immature growth plates; those aged 13–15 years old, representing the early stage of growth plate maturation; those aged 16–17 years old, representing the late stage of maturation; and those aged 18 years old and older, representing growth plate maturity similar to that of adults. The annual trends in the number of ACL reconstruction surgeries performed within one year after an ACL tear diagnosis were analyzed during the study period. The surgical cases were categorized and described based on age group, gender, and the location of the diagnosing hospital. The classification of urban and rural areas for the hospitals where patients received treatment was determined using the administrative division codes recorded in the database.

The date of an ACL tear was defined as the first recorded date associated with an ACL injury diagnosis code during the study period, and the surgery date was defined as the first recorded date of an ACL reconstruction procedure code during hospitalization. A washout period was implemented to exclude cases of ACL tears occurring prior to the study period. Patients with an ACL tear diagnosis within two years prior to the study’s start year, 2009, were excluded from the analysis. This approach minimizes the risk of misclassifying recurrent injuries as new cases and helps establish a clear temporal relationship between ACL injury and subsequent reconstruction during the study period.

### 2.3. Statistical Analysis

The Chi-square linear-by-linear association analysis was utilized to determine the statistical significance regarding trends over time in procedural volumes, age groups, and regions. We employed Poisson regression models to assess the statistical differences in surgical trends across years, age groups, and regions. The results are presented as incidence rate ratios (IRRs) with corresponding 95% confidence intervals (CIs). This approach allows for evaluating relative occurrence rates, facilitating a comprehensive understanding of the factors influencing surgical trends.

Analyses were conducted using R software version 3.4.3 (The R Foundation for Statistical Computing, Vienna, Austria) and SAS Enterprise Guide version 7.15 (SAS Institute, Cary, NC, USA).

## 3. Results

Table 1 summarizes the demographics of the patients. The total number of patients was 83,132 from 2011 to 2018. Most of the surgeries were performed on patients over 18 (90.92%). In the age group of 16 to 17, 6.46% of the patients had surgery, and in the age group of 13 to 15, 2.36% of the patients had surgery.

Table 2 lists annual trends by gender for ACL reconstruction surgery during the study period. Both gender groups showed trends of increasing the number of surgical treatments. Throughout the study period, males exhibited a markedly higher surgical frequency compared to females.

Patients within urban areas showed more surgeries than those in rural areas (Table 3), and both area groups also showed increasing trends in the number of surgeries.

**Figure 1:**
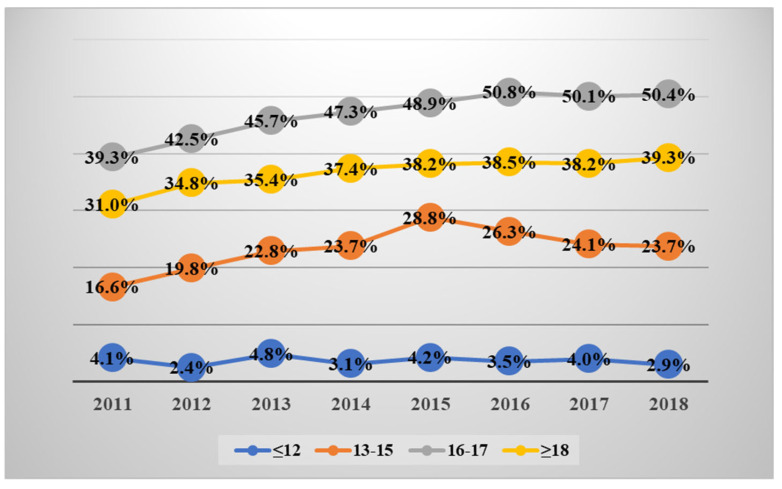
The annual number of surgeries performed within one year, categorized by age.

Annual surgical trends among age groups are listed in Table 4 and Figure 1. The age group over 13 years old showed an increasing number of surgeries from 2011 to 2018 (16.6% to 23.7% in the ≤12 years old group, 39.3% to 50.4% in the 13–15 years old group, 31.0% to 39.3% in the ≥18 years old group).

Table 5 summarizes the annual trends in surgical procedures by age group and gender. Within both gender groups, the 12 years old or younger group did not show increasing trends in ACL reconstruction surgery. In the male gender, the age group of 16–17 years old showed the greatest increasing tendency (38.9% to 51.1%); in the female gender, the age group of 13–15 years old showed the greatest increase (26.6% to 35.6%).

Results of Poisson regression for the incidence rate ratio (IRR) of each variable for the number of surgeries are summarized in Table 6. The increasing years showed significant IRR for the number of surgeries (IRR = 1.03, *p <* 0.001). The female group showed significantly less IRR than males (IRR = 0.62, *p <* 0.001). Rural areas showed significantly lesser IRR than urban areas (IRR = 0.52, *p <* 0.001). In the case of the age group, all age groups over 13 years old showed significantly greater IRR than the ≤12 year old group.

Table 7 shows the results of Poisson regression for annual surgery trends in each age group. The age group of 12 years old or younger group showed no significant change in surgical trends during the study period. All age groups over 13 years old showed significantly increasing trend of ACL reconstruction surgery during the study period (2011 to 2018, IRR = 1.04, 1.03, 1.03 in the group with 13–15 years old, 16–17 years old, group with ≥18 years old, respectively, *p <* 0.001 in all three groups)

## 4. Discussion

This study aimed to analyze the trends in ACL reconstruction surgeries over the past decade, focusing on four distinct age groups: ≤12 years, 13–15 years, 16–17 years, and ≥18 years. Utilizing Poisson regression to calculate incidence rate ratios (IRRs), we assessed the statistical significance of differences in surgical frequencies across these age groups and other factors from 2011 to 2018. Our findings indicate negligible surgeries in the ≤12 years old cohort, with minimal variation throughout the study period. Conversely, there was a notable increase in ACL reconstruction frequencies among patients under 18 years old with immature growth plates over the 8 years.

The paucity of ACL reconstructions in children aged ≤12 years old aligns with existing literature, which suggests a conservative approach due to concerns about potential growth disturbances and physeal damage [15]. This patient group with skeletal immaturity necessitates careful consideration and often leads clinicians to favor non-operative management or delayed surgical intervention until further growth is achieved [16].

In contrast, the increasing trend of ACL reconstructions in adolescents aged 13–17 years old reflects a shift in clinical practice towards early surgical intervention in this age group [17]. This change is likely influenced by a growing body of evidence supporting the safety and efficacy of ACL reconstruction in skeletally immature patients, aiming to restore knee stability and facilitate a return to pre-injury activity levels [18]. However, it is imperative to balance the benefits of early surgery against the risks of growth disturbances, necessitating individualized patient assessments [15]. In younger patients, concerns regarding growth plate (physeal) injury necessitate physeal-sparing techniques, and conservative management is often preferred to delay surgical intervention [19]. Conversely, adolescents exhibit higher rates of ACL reconstruction, driven by increased sports participation and a greater emphasis on early return to play [20]. Moreover, skeletal maturity and changes in ligamentous properties in adolescents allow for more standardized ACL reconstruction techniques, similar to those used in adults.

Our findings are consistent with global trends. A 15-year nationwide study in Italy reported a consistent increase in ACL reconstruction surgeries among patients younger than 15 years old, highlighting a significant uptick in younger populations [21]. Similarly, a bibliometric analysis indicated a growing research focus on ACL reconstruction in children and adolescents over the past two decades, reflecting heightened clinical attention to this age group of patients [18]. These parallels suggest a worldwide inclination towards addressing ACL injuries surgically in younger cohorts, possibly due to heightened sports participation and advancements in surgical techniques [22].

Despite the observed increase in surgical interventions among adolescents, it is crucial to consider the potential for higher revision rates in younger patients. Studies have identified patients under the age of 25 as a significant risk factor for revision ACL reconstruction, underscoring the need for meticulous surgical planning and robust postoperative rehabilitation protocols in this demographic [23,24]. This increased risk is likely attributed to their higher level of sports participation, greater exposure to pivoting and cutting movements, and potential deficiencies in neuromuscular control despite early return to activity [25,26]. Additionally, anatomical and biomechanical factors, including ligamentous laxity and ongoing skeletal maturation, may contribute to increased graft failure rates in this population [25].

The current study shows that trends of ACL reconstruction are clearly different between urban and rural hospitals. This seems to be mainly due to gaps in medical infrastructure, surgeon availability, and access to rehabilitation. Urban hospitals usually perform more ACL reconstruction and often use advanced techniques, such as all-inside reconstruction, because they have better access to specialized orthopedic surgeons and modern technology. Rehabilitation after the surgery is also important for good outcomes, but rural patients may have fewer opportunities to receive proper physical therapy compared to those in urban areas.

Korean national healthcare data indicate that ACL reconstruction in pediatric patients follows a more conservative trend compared to global guidelines [27]. Despite recommendations for early surgical intervention in highly active children with persistent instability, the rate of ACL reconstruction in patients aged ≤12 years showed no significant increase over time in this study, suggesting a preference for non-surgical management or delayed intervention. In contrast, ACL reconstruction rates in adolescents and adults (≥13 years old) exhibited a significant upward trend, aligning with international practices; however, the rate of increase (IRR = 1.04, *p* < 0.001) was relatively gradual. This discrepancy may be influenced by concerns over growth plate injury, differences in patient and caregiver treatment preferences, and variations in healthcare accessibility. Additionally, Korea places less focus on training young children for competitive sports compared to Western countries, which may partly explain the lower rate of early ACL reconstruction in pediatric patients.

This study has several limitations, including its retrospective design and reliance on administrative data, which may lack detailed clinical information. Additionally, while we accounted for variables such as age, gender, and hospital location, other factors like activity level, injury mechanism, and socioeconomic status were not analyzed. Future research should incorporate these variables to provide a more comprehensive understanding of the trends and outcomes associated with ACL reconstruction in pediatric and adolescent populations. Additionally, this study lacks detailed information on individual surgical techniques and outcomes. The limited number of pediatric ACL reconstruction cases makes it difficult to analyze surgical methods and prognoses comprehensively. The absence of detailed clinical data restricts understanding of surgeon preferences and patient-specific factors. Future research incorporating surgical records and long-term follow-up data are needed to better assess age-specific treatment strategies and outcomes.

## 5. Conclusions

In conclusion, our analysis reveals a stable, low frequency of ACL reconstruction surgeries in children aged ≤12 years old, with no significant change over the study period. Conversely, there is a discernible increase in surgical interventions among adolescents aged 13–17 years old, mirroring international trends. These findings underscore the evolving approach to managing ACL injuries in younger populations, balancing the imperative to restore knee function and prevent further injury against the potential risks associated with surgical intervention in skeletally immature patients.

## Figures and Tables

**Table 1:** Demographic table.

Study Duration	2011 to 2018
Operated patients number (Total number, %)	83,132 (233,478, 35.6%)
Age group (1:2:3:4, %) *	224:1960:5367:75,581 (0.3:2.4:6.5:90.9)
Sex (Male: Female, %)	66,157:16,975 (79.6:20.4)
Region (Urban: Rural, %)	81,543:1589 (98.1:1.9)
Combined procedures (Number, %)	2508 (3.02%)
Chondroplasty	2009/2508, 80.1%
Other ligament repair	499/2508, 19.9%

* Age group, (≤12: 13–15: 16–17: ≥18) years.

**Table 2:** The frequency of surgeries performed within one year after the initial diagnosis, categorized by gender.

	Male		Female	
	No.	%	No.	%
2011	7827	35.2	1850	18.6
2012	8324	38.4	2093	22.8
2013	8408	39.5	2231	23.7
2014	8553	41.0	2141	25.3
2015	8764	42.3	2106	25.5
2016	8450	42.0	2216	27.2
2017	7922	41.0	2229	27.8
2018	7909	43.0	2109	27.2

**Table 3:** The frequency of surgeries performed within one year after the initial diagnosis, based on the location of the medical facility.

	Urban		Rural	
	No.	%	No.	%
2011	9479	30.8	198	14.4
2012	10,218	34.8	199	13.4
2013	10,433	35.5	206	16.1
2014	10,498	37.2	196	18.1
2015	10,643	38.2	227	21.1
2016	10,465	38.3	201	20.7
2017	9949	38.1	202	21.2
2018	9858	38.8	160	22.0

**Table 4:** The frequency of surgeries performed within one year after the initial diagnosis, categorized by age group.

	≤12		13–15		16–17		≥18		Total	
	No.	%	No.	%	No.	%	No.	%	No.	%
2011	41	4.1	169	16.6	494	39.3	8973	31.0	9677	30.1
2012	20	2.4	211	19.8	579	42.5	9607	34.8	10,417	33.8
2013	37	4.8	252	22.8	624	45.7	9726	35.4	10,639	34.6
2014	22	3.1	263	23.7	705	47.3	9704	37.4	10,694	36.5
2015	30	4.2	327	28.8	751	48.9	9762	38.2	10,870	37.5
2016	26	3.5	290	26.3	775	50.8	9575	38.5	10,666	37.7
2017	27	4.0	228	24.1	738	50.1	9158	38.2	10,151	37.5
2018	21	2.9	220	23.7	701	50.4	9076	39.3	10,018	38.3

**Table 5:** The frequency of surgeries performed within one year after the initial diagnosis, categorized by age group and stratified by gender.

	**≤12**		**13** **–15**		**16** **–17**		**≥18**		**Total**	
**Male**										
	**No.**	**%**	**No.**	**%**	**No.**	**%**	**No.**	**%**	**No.**	**%**
2011	27	4.0	97	13.0	378	38.9	7325	36.9	7827	35.2
2012	10	1.8	128	16.5	472	44.1	7714	40.1	8324	38.4
2013	23	4.6	157	19.7	493	47.5	7735	40.8	8408	39.5
2014	13	2.5	158	20.0	548	47.2	7834	42.6	8553	41.0
2015	22	4.5	204	24.9	600	50.2	7938	43.6	8764	42.3
2016	11	2.2	170	22.1	612	51.3	7657	43.4	8450	42.0
2017	16	3.4	128	19.4	568	50.0	7210	43.0	7922	41.6
2018	12	2.5	112	17.9	565	51.1	7220	44.7	7909	43.0
**Female**										
	**No.**	**%**	**No.**	**%**	**No.**	**%**	**No.**	**%**	**No.**	**%**
2011	14	4.5	72	26.6	116	40.7	1648	18.2	1850	18.6
2012	10	3.9	83	28.7	107	36.9	1893	22.6	2093	22.8
2013	14	5.3	95	31.1	131	40.2	1991	23.4	2231	23.7
2014	9	4.4	105	33.2	157	47.9	1870	24.6	2141	25.4
2015	8	3.5	123	39.1	151	44.3	1824	24.8	2106	25.5
2016	15	5.9	120	35.9	163	48.8	1918	26.5	2216	27.2
2017	11	5.2	100	35.0	170	50.2	1948	27.1	2229	27.8
2018	9	3.9	108	35.6	136	47.6	1856	26.7	2019	27.2

**Table 6:** Poisson regression analysis for analyzing changes in surgery frequency across multiple factors.

	IRR	95% CI	*p* Value
Total			
Year	1.03	1.02–1.03	<0.001
Sex			
Male	Reference		-
Female	0.62	0.61–0.63	<0.001
Region			
Urban	Reference	-	-
Rural	0.52	0.50–0.55	<0.001
Age group			
≤12	Reference	-	-
13–15	6.03	5.25–6.93	<0.001
16–17	11.70	10.23–13.37	<0.001
≥18	9.40	8.25–10.72	<0.001

IRR, incidence rate ratio; CI, confidence interval.

**Table 7:** Changes in the number of surgeries over the entire study period using Poisson regression analysis.

	IRR	95% CI	*p* Value
≤12	0.99	0.93–1.04	0.6834
13–15	1.04	1.02–1.06	<0.001
16–17	1.03	1.02–1.05	<0.001
≥18	1.03	1.02–1.03	<0.001

IRR, Incidence rate ratio; CI, confidence interval.

## Data Availability

The access period for scholarly use of the government national database used in this study has ended. Therefore, no data are available from after the current study analysis was finished.
